# A simple and cost-saving phenotypic drug susceptibility testing of HIV-1

**DOI:** 10.1038/srep33559

**Published:** 2016-09-19

**Authors:** Yunceng Weng, Ling Zhang, Jianfeng Huang, Jin Zhao, Peifang Luo, Siyuan Bi, Zhengrong Yang, Hai Zhu, Jean-Pierre Allain, Chengyao Li

**Affiliations:** 1 Department of Transfusion Medicine, Southern Medical University, Guangzhou, China; 2 First Clinical Medicine School, Southern Medical University, Guangzhou, China; 3 Shenzhen Center of Disease Prevention and Control, Shenzhen, China; 4 Shenzhen Bioeasy Company, Shenzhen, China; 5 Department of Haematology, University of Cambridge, UK; 6 School of Public Health and Tropical Medicine, Southern Medical University, Guangzhou, China

## Abstract

It is essential to monitor the occurrence of drug-resistant strains and to provide guidance for clinically adapted antiviral treatment of HIV/AIDS. In this study, an individual patient’s HIV-1 pol gene encoding the full length of protease and part of the reverse transcriptase was packaged into a modified lentivirus carrying dual-reporters ZsGreen and luciferase. The optimal coefficient of correlation between drug concentration and luciferase activity was optimized. A clear-cut dose-dependent relationship between lentivirus production and luciferase activity was found in the phenotypic testing system. Fold changes (FC) to a wild-type control HIV-1 strain ratios were determined reflecting the phenotypic susceptibility of treatment-exposed patient’s HIV-1 strains to 12 HIV-1 inhibitors including 6 nucleoside reverse-transcriptase inhibitors (NRTIs), 4 non-nucleoside reverse transcriptase inhibitors (NNRTIs) and 2 protease inhibitors (PIs). Phenotypic susceptibility calls from 8 HIV-1 infected patients were consistent with 80–90% genotypic evaluations, while phenotypic assessments rectified 10–20% genotypic resistance calls. By a half of replacement with ZsGreen reporter, the consumption of high cost Bright-Glo Luciferase Assay is reduced, making this assay cheaper when a large number of HIV-1 infected individuals are tested. The study provides a useful tool for interpreting meaningful genotypic mutations and guiding tailored antiviral treatment of HIV/AIDS in clinical practice.

During the past decades, the numbers of HIV-1 inhibitors have been approved for the treatment of HIV-1 infected patients. The highly active antiretroviral therapy (HAART), which targeted the reverse transcriptase (RT) and/or protease of HIV-1 by combination of two or more inhibitors, was considered one of the most cost-effective therapeutic interventions for HIV-1 infected patients^1^. It had been shown not only to improve the quality of life of HIV-1 infected patients but also to reduce the risk of HIV-1 dissemination^2^,^3^. Despite their capacity of suppressing viral production and reducing the rate of virus transmission, HAART failed to eradicate the virus from HIV-1 infected patients^4^. Consequently, the clinical benefits of antiretroviral therapy had been compromised by the emergence of HIV-1 drug-resistant strains primarily due to escape virus variants under the drug selection pressure^5^. Therefore, it is essential to monitor the occurrence of drug-resistant strains and to provide guidance for clinically adapted antiviral treatment.

Currently, there are two types of genotypic and phenotypic drug resistance tests available for monitoring the HIV-1 drug resistance. The genotypic resistance test that can detect specific resistance-related mutations in the target genes of HIV-1 by DNA sequencing, is more frequently used than the phenotypic test due to ease to perform, low costs and interpretation algorithms available online^6^,^7^. However, the genotypic assay can be difficult to interpret resistance levels when it comes to sequences with unusual mutations or complex patterns of mutation combinations^8^. The phenotypic resistance test, which can detect the viral production or the enzymatic activity affected by HIV-1 inhibitors *in vitro*, has the ability to directly measure the drug resistance level or susceptibility of each inhibitor without any prior knowledge of mutations in HIV-1 strains. Therefore, it is considered significant that phenotypic susceptibility testing provides a precise guidance of drug usage for HIV-1 infected patients, especially patients exposed to antiviral drugs^9^. Usually, the phenotypic susceptibility assays are performed by culture of clinical strains. However, these assays require fresh peripheral blood mononuclear cells (PBMCs) from donors, are labor-intensive and time-consuming^10^. Recently, several assays either based on a recombinant virus with single-loop infection^11^,^12^,^13^, or infectious particles with multiple cycles of replication were developed^14^,^15^. Although those assays show great potential in phenotypic drug susceptibility testing, their cost remains high due to the expensive Bright-Glo Luciferase Assay System and concerns regarding bio-safety.

In this study, we developed a novel lentivirus-based phenotypic assay with two reporters: luciferase and ZsGreen. The main advantage of dual reporters is that the viral response to HIV-1 inhibitors is easily observed either by cells stained with ZsGreen under the Inverted Fluorescence Microscope 60 h after virus infection or by quantification of luciferase activity. In addition, the HIV-1 based lentivirus being known powerful and safe^16^,^17^ is an ideal vector for HIV-1 drug resistance testing. Using this new assay, the phenotypic susceptibility level of HIV-1 in infected patients was measured and analyzed against a panel of antiviral drugs available in China.

## Results

### A novel recombinant lentivirus system presenting phenotypic drug susceptibility of HIV-1

**Figure 1:**
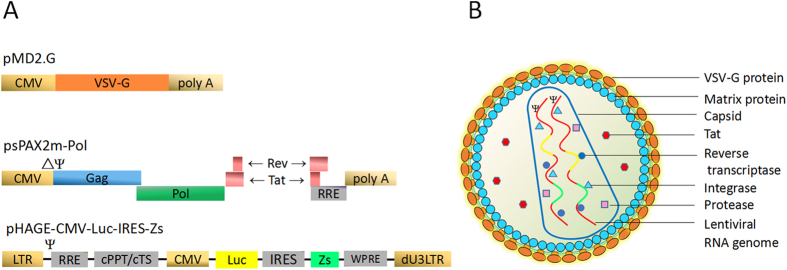
Generation of patient’s HIV-1 derived lentiviral particles with dual reporters. (**A**) A mixture of packaging plasmid psPAX2m-Pol, envelop plasmid pMD2.G and transfer plasmid pHAGE-CMV-Luc-IRES-ZsGreen were co-transfected into 293FT cells. CMV, Cytomegalovirus Promoter; VSV-G, Vesicular Stomatitis Virus G protein; Ψ, packaging signal of lentivirus; ΔΨ, packaging signal deleted; Gag, gag polyprotein that the processed proteins includes matrix protein and capsid; Pol, pol polyprotein that the processed proteins includes protease, integrase and reverse transcriptase; Tat, tat protein; Rev, rev protein; RRE, the Rev response element which allows for Rev-dependent mRNA export from the nucleus to cytoplasm; 5′-LTR, 5′ long terminal repeat; cPPT/cTS, central polypurine tract and central termination sequence; Luc, luciferase from pGL-3 promoter vector; IRES, internal ribosome entry site; Zs, ZsGreen protein; WPRE, woodchuck posttranscriptional regulatory element; dU3LTR, self-inactivating 3′ long terminal repeat. (**B**) Structure of lentiviral particles produced by co-transfection of three plasmids into 293FT cells.

The modified lentivirus packaging system consisted of three plasmids with envelope, packaging and transfer functions carrying a patient’s HIV-1 pol gene and dual reporter genes of luciferase and ZsGreen (Supplemental Figs S1 and S2). The patient’s HIV-1 pol gene within packaging plasmid psPAX2m-Pol was integrated into recombinant lentiviral genome with envelop plasmid pMD2.G and transfer plasmid pHAGE-CMV-Luc-IRES-ZsGreen in 293FT cells (Fig. 1). The patient’s HIV-1 derived lentivirus could functionally present the drug susceptibility by luciferase or ZsGreen reporters in the transduced or infected 293A cells in the presence of different concentrations of antiviral drugs.

### Establishment of optimized MOI, cell density and time point in phenotypic drug susceptibility test

**Figure 2:**
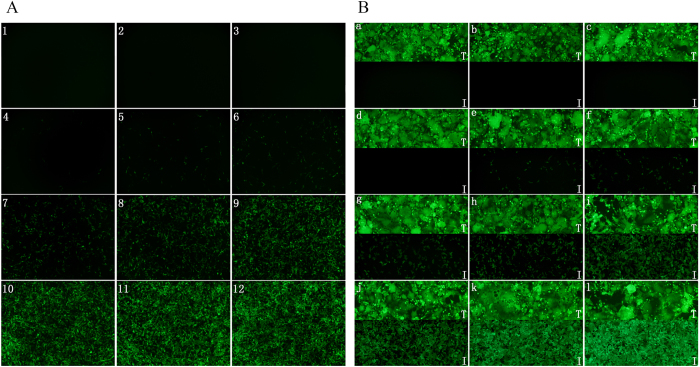
Selection of suitable range of antiviral drug concentration by ZsGreen reporter. (**A**) Effect of serial dilution of Zidovudine (a representative of NRTIs and NNRTIs) on protein expression of lentivirus infected 293A cells. 1, 10 MOI + 333.333 μM/L; 2, 10 MOI + 111.111 μM/L; 3, 10 MOI + 37.037 μM/L; 4, 10 MOI + 12.345 μM/L; 5, 10 MOI + 4.115 μM/L; 6, 10 MOI + 1.371 μM/L; 7, 10 MOI + 0.457 μM/L; 8, 10 MOI + 0.152 μM/L; 9, 10 MOI + 0.0508 μM/L; 10, 10 MOI + 0.0169 μM/L; 11, 10 MOI + 0.0056 μM/L; 12, 10 MOI. (**B**) Effect of serial dilution of Lopinavir (a representative of PIs) on lentivirus production. a, 1371 nM/L; b, 457 nM/L; c, 152 nM/L; d, 50.8 nM/L; e, 16.9 nM/L; f, 5.6 nM/L; g, 1.88 nM/L; h, 0.627 nM/L; i, 0.209 nM/L; j, 0.06969 nM/L; k, 0.02323 nM/L. T, Transfection of 293FT cells with 3 plasmids of packaging system in the presence of different concentration of drug; I, Infection of 293A cell with 50 μl lentivirus supernatant from the paired Transfection.

In order to establish optimal assay conditions, the multiplicity of infection (MOI), cell density and time point of detection were optimized with a representative HIV-1 inhibitor in phenotypic susceptibility testing. First, a titer of patient’s HIV-1 derived lentivirus was determined within a range of virus dilution folds around 10% ZsGreen positive rate from lentivirus-transduced 293A cells. Secondly, the lentivirus with ZsGreen reporter was used for selecting suitable drug dilution range and pre-titration of the MOI (Fig. 2), in which the drug dilutions corresponding to 10–90% ZsGreen positive cells were primarily chosen for further titration of MOI by the lentivirus with luciferase reporter. When the concentration of Zidovudine (a representative of NRTIs and NNRTIs) decreased in the presence of lentivirus transduced 293A cells, the positive rate of ZsGreen cells increased (Fig. 2A), in which the drug destroyed the function of HIV-1 pol and inhibited the expression of ZsGreen in the virus infection process. In a different mechanism from NRTIs or NNRTIs, when the concentration of Lopinavir (a representative of PIs) decreased in the presence of lentiviral packaging process in the cells (Fig. 2B–T), the positive rate of ZsGreen cells increased since Lopinavir prevents maturation of the internal structural proteins and of the viral enzymes in a dose-dependent manner.

**Figure 3:**
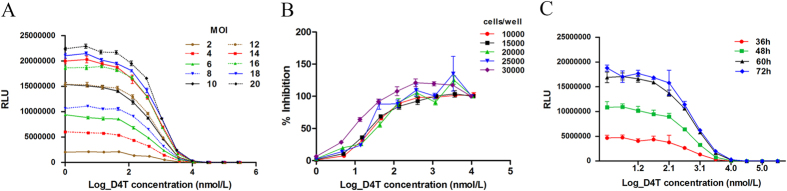
Optimization of MOI, cell density and detection time of phenotypic susceptibility testing. (**A**) Determination of the optimal MOI. (**B**) Determination of the optimal cell density. (**C**) Optimization of detection time. An antiviral drug D4T was used and the D4T concentrations are presented in the log scale. RLU, Relative luciferase activity unit.

The luciferase activity increased from 2 to 20 MOI (Fig. 3A), the optimal coefficient of correlation (R^2^ = 0.958, *P* < 0.01) between drug concentration and luciferase activity being 10 MOI. Meanwhile, between five different cell densities tested (1 × 10^4^, 1.5 × 10^4^, 2 × 10^4^, 2.5 × 10^4^ and 3 × 10^4^ cells/well), the highest R^2^(0.998, P < 0.01) was observed with 1.5 × 10^4^ cells/well (Fig. 3B). In addition, the optimal detection time was 60 h after lentivirus inoculation since not only it gave the highest R^2^(0.938, P < 0.01) between drug concentration and luciferase activity, but also the second highest activity of luciferase (Fig. 3C). Finally, stability and reproducibility of the phenotypic resistance testing were evaluated by determining the concentration of 12 antiviral drugs at 50% inhibition (IC_50_) of lentivirus infection in quadruplicate from three separate tests (Table 1). The data appeared highly reproducibility and stability with coefficient of variation (CV) below 15%.

### Analysis of genotypic drug resistance-associated mutations from patient’s HIV-1 strains

The HIV-1 infected patient information was indicated in Table 2, showing that all patients were previously treated with a combination of antiviral drugs. All eight patients had CD4 cell counts below 400 cells/mm^3^ and five of them below 200 cells/mm^3^. Patient’s HIV-1 RNA load ranged between 155 and 104000 copies/ml. To analyze the drug resistance associated with viral mutations from these patients, the HIV-1 pol genes were amplified and sequenced. Based on the genotypic resistance database (the Stanford University HIV Drug Resistance Database) available for 10 HIV-1 inhibitors involved in this study, the mutations from those HIV-1 strains were associated with genotypic drug resistance to NRTIs, NNRTIs and PIs, respectively (Table 3). The antagonistic mutations K65R and M184V were found from two patients ID 38290075 and 38290022, respectively (Table 3). Mutation K65R from the patient ID 38290075 was previously reported susceptible to zidovudine (AZT) and stavudine (D4T), but decreased the susceptibility of HIV-1 to tenofovir, didanosine, abacavir and lamivudine^18^. Mutation M184V from the patient ID 38290022 was described partly susceptible to tamoxifen (TAM), zidovudine, stavudine and tenofovir, but increased the sensitivity of HIV-1 to these antiviral agents^19^. When however, it comes to analyze the drug resistance of HIV-1strains which carried such mutations, they might be predicated resistant by genotypic resistance testing, while the phenotype of drug resistance were actually differed. Therefore, without massive correlative information of genotypic and phenotypic resistance testing of various viral mutants to individual drugs, the genotype testing was not sufficient to predict the drug resistance levels from the viral replication effect of complex mutations as well as new mutations of HIV-1 strains.

### Detection of phenotypic drug susceptibility from patients’ HIV-1 strains

Eight HIV-1 strains were measured for drug susceptibility using the recombinant lentivirus system with dual reporters (Supplemental Fig. S3). A wildtype lentivirus whose gag and pol genes are derived from the subtype B strain served as control for calculation of the fold changes (FC). The phenotypic drug susceptibility or resistance level to 12 antiviral drugs are shown in Table 4. Comparing with genotypic resistance analysis available for 10 drugs from the Stanford University HIV Drug Resistance Database (http://hivdb. stanford.edu/), the majority of phenotypic testing calls were concordant with genotypic assessments (Table 4). Approximately 21% (17/80) discrepancy rate was observed between phenotypic and genotypic drug resistance testing from these HIV-1 strains, of which 10% (8/80) drug resistance calls clearly differed by nearly two levels among susceptibility (S), low (L), middle (M) and high (H) resistance levels between two assays (Table 4). For instance, the patient’s HIV-1 strain 38290022 was susceptibility to DDI and D4T drugs and high resistance to RPV by phenotype, but middle or high resistance to DDI and D4T and low resistance to RPV by genotype, while HIV-1 strain 38290312 was highly resistant to ETR by phenotype but low resistance by genotype. The results suggested that the newly developed phenotypic susceptibility testing could rectify at least 10% of incorrect calls regarding drug resistance levels predicted by genotypic testing.

## Discussion

Drug resistance level or susceptibility testing is considered an important issue for the management of newly HIV-1 infected and treatment-exposed HIV-1 infected patients^9^,^20^. When a patient was newly diagnosed with HIV-1 treatment failure, drug susceptibility testing is recommended by the US Department of Health and Human Services (DHHS), International AIDS Society (IAS-USA) and European guidelines^21^. Phenotypic susceptibility assay is optimal to provide accurate and quantitative assessments of HIV-1 strains susceptible to a large number of antiviral drugs, especially for patients receiving antiretroviral for years^9^,^22^,^23^. The principle of phenotype testing is to quantify *in vitro* viral replication in the presence of serial dilutions of antiviral drugs. Drug resistance level (susceptibility) is estimated as FC calculated from a ratio of the IC_50_ of the patient strain to the IC_50_ of a wild-type control virus.

Several phenotypic assays have been previously described for assessing HIV-1 drug resistance in clinical conditions, including the first-generation Antivirogram and PhenoSense assays^14^,^24^,^25^, the ExaVir^TM^ Drug assay^26^,^27^, the modified assays with a single cycle system or single-loop infection^11^,^12^,^13^, with two round infection^28^ or multiple cycles of replication^15^. In our study, a second-generation lentivirus vector carrying individual patient’s HIV-1 pol gene was constructed, in which the phenotypic drug-resistance was represented by dual-reporters in a single cycle of genetically modified lentivirus infected cells. This phenotypic assay has the advantages of simple, cost and time-saving and easy performing for detection of drug susceptibility to HIV-1 in a large panel of antiviral drugs.

By using the established lentivirus system, the susceptibility phenotypes to 12 antiretroviral drugs of eight treatment-exposed patients’ HIV-1 strains were assessed against a control wild-type HIV-1 strain. The modified lentiviral vector with single restriction sites of Apa I and Age I appeared to be universally capable of integrating a drug-targeting pol gene from individual HIV-1 strains. The phenotypic susceptibility of HIV-1 to inhibitors on lentivirus-infected cells was reflected by the rate of ZsGreen positive cells observed with an inverted fluorescence microscope or by flow cytometric analysis. In our pre-testing, serial wells covering 10% to 90% of the ZsGreen positive cells observed with an inverted fluorescence microscope were selected for further measurement of luciferase activity with a micro-well plate reader. By narrowing down the range of percentage of ZsGreen full positive or negative wells, the consumption of high cost Bright-Glo Luciferase Assay is reduced in half, making this assay cheaper when a large number of HIV-1 infected individuals are tested. Moreover, the fold-changes of the patient-derived lentiviruses in the presence of HIV-1 inhibitors were quantified in drug susceptibility phenotype, which could precisely guide the antiviral treatment by identifying a combination of susceptibility or low resistance drugs in clinical practice. Similarly, by using ZsGreen reporter alone, the density of green fluorescence of patient-derived lentivirus infected cells was measured by flow cytometry. The phenotypic drug susceptibility of HIV-1 strain could be evaluated by the FC against a wildtype HIV-1 control. However, the expensive flow cytometric machine was required for reading the signal of ZsGreen reporter, which might limit assay’s utility.

High reproducibility of the IC_50_ from 12 antiviral drugs was found by the new assay, with coefficient of variation (CV) ranging between 0.5% and 15%. In this study, phenotypic susceptibility testing results were consistent with 80–90% genotypic interpretations obtained from the Stanford University drug resistance database^29^, as reported in previous studies^13^,^30^. However, approximately 10–20% discrepant resistance calls from 10 drugs were found between the two assays^31^,^32^. Those discrepancies might be related to three major reasons. Firstly, many drug resistance mutations obtained by sequencing arise in complex patterns including the most common variants, which make it difficult for interpreting the correlation with HIV-1 susceptibility phenotype^33^. For example, mutation M41L is a thymidine analog mutation (TAM) that usually occurs together with T215Y and confers high-level resistance to D4T and AZT and middle-level resistance to DDI and ABC, respectively^34^. In our study, however, M41L was not accompanied with T215Y but with T215F in patient ID 38290022 (Table 4), while the phenotype of this virus appeared susceptible to DDI and D4T (FC < 3) and remained moderately resistant to ABC (FC = 6- < 10), suggesting that phenotypic testing was more suitable to evaluate drug susceptibility than genotypic testing of resistance. Secondly, presence of antagonistic mutations may explain the discrepancy of resistance between genotypic and phenotypic assays. For example, M184V mutation is reported partly to reverse resistance to TAM, AZT, D4T and TDF, and increases susceptibility of HIV-1 to these inhibitors^12^,^19^. Thus, HIV-1 strains (ID 38290022 and ID 38290063) carrying these mutations may be categorized as phenotypic susceptibility but genotypic resistance. In addition, revertant mutations T215S/C/D/E/I/V which usually do not reduce the NRTI susceptibility are considered transitions between wild-type and T215Y/F mutations, of which T215T/F mutations cause intermediate/high-level resistance to AZT and D4T respectively^35^. Speculatively, HIV-1 strains (ID 38290312) carrying T215S/C/D/E/I/V rather than T215T/F mutations are considered low-level resistance to AZT and D4T by genotypic analysis, while their phenotypic resistance testing may appear to be sensitive. Thirdly, the baseline fold change in IC_50_ may affect the determination of phenotypic resistance level or susceptibility evaluation, of which three cut-offs of technical, biological and clinical evaluations are currently used but none of them is considered fully optimal^6^,^9^,^31^,^32^. In our study, the fold changes (FC) were clearly defined as <3 indicative of susceptibility, 3- <6 indicative of low-level resistance, 6- <10 indicative of intermediate or middle-level resistance and ≥10 indicative of high-level resistance, respectively, on the basis of combining criteria described previously^6^,^31^,^36^. Despite a single patient (ID 38290075) resistant to both NRTIs and NNRTIs and moderately susceptible to PIs observed in this study, the other seven patient’s HIV-1 drug susceptibility or resistance levels were well classified for the three categories of antiviral drugs. Such information would be greatly beneficial for clinicians having to design an optimal combination of anti-HIV-1 drugs for tailored treatment in clinical practice.

In conclusion, the study developed a simple and accurate phenotypic testing assay based on the second-generation lentivirus with dual reporters, which facilitated the evaluation of HIV-1 susceptibility or resistance levels to antiviral drugs. In comparison with the conventional phenotypic assays, this testing is cheap, rapid and accurate for quantitative assessment of drug resistance levels of HIV-1 strains to antiviral inhibitors and provides a tool for interpreting the meaningful genotypic mutations and guiding the precise antiviral treatment of HIV/AIDS in clinical practice.

## Materials and Methods

### Patient samples and antiviral drugs

Plasma samples from HIV-1 infected patients were provided by the center of disease prevention and control (CDC), Shenzhen, China. All patients had been treated with antiviral drugs in the past years. All participating patients signed an informed consent for sample collection and testing. This study was approved by the Medical Ethics Committee of Southern Medical University (permit numbers: NFYY-2008-045), Guangzhou, China. All experiments were carried out in accordance with the approved guidelines.

Twelve HIV-1 inhibitors were used in this study, including six nucleoside reverse-transcriptase inhibitors (NRTIs): Didanosine (DDI), Stavudine (D4T), Zidovudine (AZT), Zalcitabine (DDC), Emtricitabine (FTC), Abacavir Sulfate (ABC), four non-nucleoside reverse transcriptase inhibitors (NNRTIs): Nevirapine (NVP), Etravirine (ETR), Dapivirine (DPV), Rilpivirine (RPV), and two protease inhibitors (PIs): Nelfinavir (NFV) and Lopinavir (LPV), which were purchased commercially from Selleckchem (Shanghai, China).

### Isolation of drug-targeting genes of HIV-1

Viral RNA was extracted from HIV-1 infected patient’s plasma samples with High Pure Viral Nucleic Acid Kit according to the manufacturer’s instructions (Roche, Pleasanton, USA). The HIV-1 pol genes encoding protease 1-99 amino acids and reverse transcriptase 1-314 amino acids were amplified by RT nested-PCR using PrimeScript^TM^ II High Fidelity RT-PCR Kit (TaKaRa, Dalian, China). The primer sets were as follows^13^: Outer-F, 5′-GCAAGAGTTTTGGCTGAAGCAATGAG-3′; Outer-R, 5′-CCTTGCCCCTGCTTCTGTATTTCTGC-3′; Inner-F, 5′-TGCAGGGCCCCTAGGAAAAAGGGCTG-3′ (Apa I); Inner-R, 5′-CACTCCATGTACCGGTTCTTTTAGAATCTC-3′ (Age I). The inner primers had added restriction sites ApaI and AgeI, respectively. The first-round PCR was done at 45 °C for 30 min and 94 °C for 2 min followed by 30 cycles of 98 °C for 10 s, 55 °C for 15 s and 68 °C for 2 min. The second-round PCR was performed at 98 °C for 5 min followed by 30 cycles of 98 °C for 10 s, 51 °C for 15 s and 68 °C for 2 min. The patient-derived PCR products were purified by an AxyPrep DNA Gel Extraction Kit (AXYGEN, Suzhou, China) and sequenced by a commercial company (Invitrogen, Guangzhou, China). The sequenced PCR fragments were digested with ApaI and AgeI, and were ready for cloning and expressing in the recombinant lentiviral vector system.

### Recombinant lentiviral vector system with three plasmids carrying dual reporter genes

Packaging plasmid psPAX2m-Pol carried the patient HIV-1-derived pol gene. Plasmid psPAX2 (# 12260) was a gift from Dr Didier Trono (Lausanne, Switzerland), which contained the pol genes from a wild-type HIV-1 subtype B NL4.3 strain (GenBank access number AF324493.2)^37^. In order to insert the drug resistance gene fragment (pol) of HIV-1 strains into the packaging plasmid psPAX2 at restriction sites Age I and Apa I, the vector was genetically modified for dysfunction of two extra sites of Age I (position nt 7697) and Apa I (position nt 863) by amplification and site-directed mutagenesis (Supplemental Table S1), designated as psPAX2m. The targeted drug resistance genes (which corresponds to the consensus sequences) from HIV-1 infected patients were individually cloned into the psPAX2m at Age I and Apa I restriction sites, which were designated as psPAX2m-Pol and were used for recombinant lentivirus production (Supplemental Fig. S1).

Envelop plasmid pMD2.G. The vector (plasmid #12259) was a gift from Didier Trono (Lausanne, Switzerland).

Transfer plasmid pHAGE-CMV-Luc-IRES-ZsGreen contained dual-reporter genes. The CMV promoter appeared to initiate higher transduction efficiency than EF1α promoter in 293A cells^38^. In order to get a stronger signal of luciferase or ZsGreen, the EF1α promoter in the pHAGE-EF1α-IRES-ZsGreen (a gift from Jeng-Shin Lee, Harvard Medical School, USA) was replaced by the CMV promoter pMD2.G. Briefly luciferase gene fragment from pGL3 Luciferase Reporter Vector (Promega, Beijing, China) was sub-cloned into the pHAGE-CMV-IRES-ZsGreen next to the IRES. The transfer vector with dual-reporters was designated pHAGE-CMV-Luc-IRES-ZsGreen and suitable for lentivirus production (Supplemental Fig. S2).

### Recombinant lentivirus production and titration

A total of 3 × 10^6^ 293FT cells were plated in 75 cm^2^ cell culture flask 36 h prior to transfection. When 75% cell confluence was reached, cells were co-transfected with a total of 15 μg of three plasmid DNAs (2.8 μg envelop plasmid pMD2.G, 5.2 μg packaging plasmid psPAX2m-Pol and 7 μg transfer plasmid pHAGE-CMV-Luc-IRES-ZsGreen) and 45 μl of X-tremeGENE HP DNA Transfection Reagent (Roche, USA) diluted in 1.5 ml of Opti-MEM medium and incubated at room temperature for 15 min. After cell culturing for 48 h at 37 °C, the lentivirus-containing supernatant was harvested after centrifugation at 3000 rpm at 4 °C for 20 min and filtration with a Millex-HV 0.45 μm filter (Millipore, USA).

Lentivirus titration was performed as a previously described assay with modifications^39^. Briefly, a total of 5 × 10^5^ 293A cells were seeded in 6-well plate (Corning, USA), and cultured for 24 h. The cells were inoculated in a series of lentivirus and incubated for 12 h. The cell medium was changed completely, replaced with fresh medium, and further incubated for 48 h. Cells in each well were counted by flow cytometry in order to enumerate the rate of ZsGreen positive cells. The titer was determined by the following formula: (F × C/V) ×D. F = frequency of ZsGreen-positive cells (the percentage obtained and divided by 100), C = total number of cells in the well at the time of infection, V = volume of lentivirus diluent in ml, D = lentivirus dilution fold. Following the formula and standardizing the results, lentivirus dilution fold was determined with a range of 1–30% GFP-positive cells observed after viral transduction.

### Optimization of phenotypic drug susceptibility test

The lentivirus produced by co-transfection with psPAX2m, pMD2.G and pHAGE-CMV-Luc-IRES-ZsGreen served as a wild-type HIV-1 control of phenotypic resistance since the pol gene within psPAX2m was derived from a wild-type HIV-1 subtype B NL4.3 strain susceptible to HIV-1 inhibitors. To see whether the HIV-1 inhibitor affected the viral infection and protein production, Zidovudine (D4T) was used as a representative antiviral drug for optimizing the testing conditions according to a previous study^12^. The multiplicity of infection (MOI), cell density and time points were optimized to obtain the most suitable luciferase activity in the test. The luciferase activity was measured using the Bright-Glo Luciferase Assay System (Promega, USA) in a micro-well plate reader (Wallik 1420, Perkin Elmer, USA). Each data point was tested in quadruplicate. The susceptibility of HIV-1 pseudotyped lentivirus to those 12 antiviral drugs was determined at three different time points, and the reproducibility of testing was evaluated.

### Phenotypic drug susceptibility testing for patient’s HIV-1 strains

The amplified HIV-1 pol gene sequences were submitted to the Stanford HIV Drug Resistance Database. The genotypic drug resistance of HIV-1 from the treated patients was analyzed with the HIVdb program available from the Stanford University HIV Drug Resistance Database^29^. Based on the aforementioned results, phenotypic drug susceptibility testing for patient’s HIV-1 samples was performed using the newly developed lentivirus-based assay. A dose of 10 × MOI of recombinant lentivirus with an individual targeting HIV-1 pol gene in the presence of different concentrations of NRTIs or NNRTIs were applied to infect 1.5 × 10^4^ 293A cells per well within 96 wells of cell culture plate in the test, and then the activity of luciferase was determined 60 h post-infection. All antiviral drug testing were performed in quadruplicate each test result being derived from three representative experiments.

The antiviral effect of protease inhibitors (PIs) was measured during viral production. Briefly, 4 × 10^4^ 293FT cells/100 μl were seeded in each well of 96-well plate and incubated for 36 h. The cells were transfected with 15 μl of mixture with three plasmid DNAs in Opti-MEM and X-tremeGENE HP DNA Transfection Reagent (Roche, USA), and then 8 h later the cell culture was changed with 100 μl of fresh medium containing a 3-fold serial concentrations of PIs. After 48 h of incubation of the transfected cells with PIs, cells were spun down by centrifugation at 4000 rpm for 20 min at 4 °C. Fifty μl of virus-containing supernatant were added to the 24 h pre-seeded 293A cells for measuring the activity of luciferase after 60 h incubation from lentivirus transduced cells, respectively.

### Data analysis

The percentage of inhibition was calculated using the following formula^12^: Viral inhibition rate (%) = (1- RLU in the drug treated group/RLU in the control) ×100% (RLU, relative luciferase activity unit). The drug concentration producing 50% inhibition (IC_50_) on an individual HIV-1 strain was calculated using nonlinear regression analysis. The phenotypic resistance level of a detected HIV-1 strain to an individual antiviral inhibitor was expressed as a fold-change (FC), the ratio of the IC_50_ of the tested strain to the IC_50_ of a wild-type strain control^34^. The adjusted criteria for classifying the phenotypic resistance levels (susceptibility) of antiviral drugs to HIV-1 strains were defined as a ratio of FC < 3 indicative of drug susceptibility (S), FC = 3- <6 indicative of low drug resistance (L), while FC = 6- <10 and FC ≥ 10 indicative of intermediate or middle (M) and high drug resistance (H), respectively^6^,^31^,^36^. All analyses were done with the software SPSS version 21.0.

## Additional Information

**How to cite this article**: Weng, Y. *et al*. A simple and cost-saving phenotypic drug susceptibility testing of HIV-1. *Sci. Rep.* **6**, 33559; doi: 10.1038/srep33559 (2016).

## Supplementary Material

Supplementary Information

## Figures and Tables

**Table 1:** Reproducibility of phenotypic resistance test for 12 antiviral drugs.

Drug	Concentration of drug at IC_50_ (μmol/L or nmol/L*)			Mean (μmol/L or nmol/L*)	SD	CV (%)
	1	2	3			
Didanosine (DDI)	46.751	44.797	47.904	46.484	1.5706142	3.38
Stavudine (D4T)	0.735	0.794	0.780	0.769667	0.0308275	4.01
Zidovudine (AZT)	0.212	0.243	0.266	0.240333	0.0270986	11.28
Zalcitabine (DDC)	0.363	0.347	0.378	0.362667	0.0155027	4.27
Emtricitabine (FTC)	0.291	0.296	0.289	0.292000	0.0036056	1.23
Abacavir Sulfate (ABC)	1.227	1.242	1.123	1.197333	0.0648100	5.41
Nevirapine (NVP)	0.121	0.122	0.121	0.121333	0.0005774	0.48
Etravirine (ETR)*	2.469	2.779	2.190	2.4793	0.2946	11.88
Dapivirine (DPV)*	3.502	2.740	3.067	3.103	0.3822	12.32
Rilpivirine (RPV)*	3.241	3.945	4.365	3.850	0.5679	14.75
Nelfinavir (NFV)*	0.280	0.226	0.280	0.262	0.0311769	11.9
Lopinavir (LPV)*	0.04694	0.04567	0.04011	0.04424	0.0036326	8.2

IC50, the drug concentration producing 50% inhibition of positive ZsGreen cells; SD, standard deviation; CV, coefficient of variation. *The concentration of these drugs is presented in nmol/L. The data were obtained from three representative experiments in quadruplicate.

**Table 2:** Clinical information of HIV-1 infected patients.

Patient ID	Date of treatment start	CD4 count (cells/mm^3^)	Viral load (copies/ml)	Antiviral drugs used for treatment
38290022	2004-10-15	124	2740	D4T, DDI, NVP^1^
38290063	2006-06-05	112	7130	D4T, DDI, NVP
38290075	2006-12-15	377	208	D4T, DDI, NVP
38290079	2006-12-22	244	104000	D4T, DDI, EFV
38290243	2009-02-16	214	2290	AZT, 3TC, EFV
38290306	2009-07-02	176	155	D4T, 3TC, NVP
38290309	2009-07-09	108	321	AZT, 3TC, NVP
38290312	2008-03-03	26	87500	AZT, 3TC, EFV

^*^For full names, see Table 1.

**Table 3:** Analysis of drug resistance-associated mutations in HIV-1 strains from 8 patients.

Patient ID	NRTI resistance mutations	NNRTI resistance mutations	Other RT mutations	PI major resistance mutations	PI minor resistance mutations	Other PI mutations
38290022	M41L, M184V, T215F	K101H, V106A, G190A, F227L	K20R, V60I, A98S, D123E, I135V, I178L, V179I, G196E, T200A, Q207K, R211V, K223Q, L228R, A272P, K275R, V276I			D60E, I62V, L63P, I72V, V77I, I93L
38290063	D67N, T69D, K70R, M184V, T215F, K219Q	K103N, G190A	K101Q, K122E, D123N, D177N, I178M, T200A, Q207E, R211K, E248D		A71T	N37T, R41K, Q61E, I62V, L63P, I93L
38290075	K65R, K70R, V75I, F77L, Y115F, F116Y, Q151M, K219E	K103N, Y181C	S68G, T69I, R172K, I178M, T200I, R211K, L228R, V245E, A272P, E297K, Y318N,			I15V, R41K, D60E, L63P
38290079		K103N	E6D, K11T, V35T, T39K, K43E, S68G, K122E, D123N, K166R, K173I, Q174E, D177E, I178M		L10I	I15V, K20R, E35D, M36I, N37D, R41K, I62V, L63P, H69K
38290243		V90I, K101E, E138K	E6P, K11R, K102N, D177N, I178M, R211K, V245E, A272P, T296S, E297K			T4S, I15V, N37S, R41K, D60E, L63P
38290306		K103N, Y181C, P225H	K11R, K102R, D123E, T200I, R211K, V245E, A272P, P294S, T296S, E297K			I15V, M36I, N37S, P39Q, R41K, K45R, D60E, L63P
38290309			E6D, V35I, G99X, K102S, I135V, I178, G196E, T200E, E204K, L205M, Q207E, A272P, I293V, E297K		A71V	Y59F, L63S, K70T, I72T, V77I, I93L
38290312	T215S	Y188L	E6D, I135T, Q197E, R211K, I244V, G262E, A272S, K277R, A288S, I293V, E297K	L90M	L10I, A71T	I13V, K20L, E35D, M36I, I64V, V77I, I93L

The genotypic drug resistance of HIV-1 strains to the study involved five NRTIs of Didanosine, Stavudine, Zidovudine, Emtricitabine, Abacavir Sulfate and three NNRTIs of Nevirapine, Etravirine and Rilpivirine was available from the Stanford University HIV drug resistance database (http://hivdb. stanford.edu/; accessed on April 28, 2014 and February 20, 2016).

**Table 4:** Comparison of phenotypic and genotypic drug resistance testing of HIV-1 strains.

		Level of drug resistance											
HIV-1 strain	Type	DDI	D4T	AZT	DDC	FTC	ABC	NVP	ETR	DPV	RPV	NFV	LPV
38290022	Phenotypic	1.22	1.6	17.7	1.38	>1000	6.7	>1000	5	7.85	12.2	0.34	0.9
	Genotypic	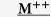	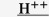	H	NA	H	M	H	L	NA	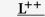	S	S
38290063	Phenotypic	2.38	2	14.3	2.09	>1000	3.95	>1000	3.6	3.86	7.2	0.26	1.17
	Genotypic	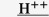	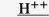	H	NA	H	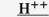	H	L	NA	L	S	S
38290075	Phenotypic	>1000	51	>1000	74	>1000	97	>1000	645	>1000	215	2.98	1.57
	Genotypic	H	H	H	NA	H	H	H	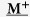	NA	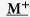	S	S
38290079	Phenotypic	1.2	0.74	0.48	0.87	0.35	0.94	28	1.6	1.69	1.2	0.29	0.93
	Genotypic	S	S	S	NA	S	S	H	S	NA	S	S	S
38290243	Phenotypic	1.1	1	0.4	1.75	1.85	0.8	4.23	15	8.83	7.32	1.04	2.46
	Genotypic	S	S	S	NA	S	S	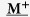	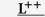	NA	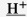	S	S
38290306	Phenotypic	0.92	0.86	0.3	0.7	0.66	0.8	>1000	87	270	30.74	2.25	2.1
	Genotypic	S	S	S	NA	S	S	H	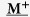	NA	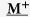	S	S
38290309	Phenotypic	1.07	1	1.4	1.07	0.97	0.74	0.5	2.7	1.37	2.08	1.35	2
	Genotypic	S	S	S	NA	S	S	S	S	NA	S	S	S
38290312	Phenotypic	1.02	0.68	0.95	0.6	0.89	1.15	>1000	17.8	48.4	16.29	10	0.42
	Genotypic	S	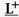	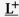	NA	S	S	H	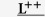	NA	H	H	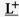
Wild type	Phenotypic	1	1	1	1	1	1	1	1	1	1	1	1
	Genotypic	S	S	S	NA	S	S	S	S	NA	S	S	S

NA, not available. Genotypic drug resistances are indicated by S (susceptible), L (low resistance), M (middle resistance) or H (high resistance) according to the database [Available at: http://hivdb. stanford.edu/; accessed on April 28, 2014 and February 20, 2016], of which capital letters in bold with “+” or “++” and underline indicate discrepancies between phenotypic and genotypic resistance for at least one or two levels, respectively. Phenotypic drug resistance is presented as the fold change (FC), of which FC < 3 indicates susceptibility, FC = 3- < 6 low resistance, FC = 6- < 10 middle resistance, FC ≥ 10 high resistance of drug to HIV-1 strains, respectively^6^,^31^,^36^.
