# Supplementary material for: A simple and cost-saving phenotypic drug susceptibility testing of HIV-1
Source: Sci Rep. 2016 Sep 19;6:33559. doi: 10.1038/srep33559 (PMC5027539; doi:10.1038/srep33559)

**Supporting Information**

# A simple and cost-saving phenotypic drug susceptibility testing of HIV-1

Yunceng Weng [1], Ling Zhang [1*], Jianfeng Huang [2], Jin Zhao [3], Peifang Luo [1], Siyuan Bi [4], Zhengrong Yang [3], Hai Zhu [4], Jean-Pierre Allain [1,5], Chengyao Li [1,6*]

[1] Department of Transfusion Medicine, Southern Medical University, Guangzhou, China;

[2] First Clinical Medicine School, Southern Medical University, Guangzhou, China;

[3] Shenzhen Center of Disease Prevention and Control, Shenzhen, China;

[4] Shenzhen Bioeasy Company, Shenzhen, China;

[5] Department of Haematology, University of Cambridge, UK;

[6] School of Public Health and Tropical Medicine, Southern Medical University, Guangzhou, China;

**Corresponding**: Chengyao Li, PhD, or Ling Zhang, PhD, Department of Transfusion Medicine, Southern Medical University, Guangzhou 510515, China. Email: chengyaoli@hotmail.com (CL); or zhangling1982@163.com (LZ)

**Supplemental Table S1. Primers used for modification of psPAX2.**

| Name | Sequence (5'-3') | |
|---|---|---|
| AgeI-F | CGAA*GAGCTC*ATCAGAACAGTCAGACTCATCAAGCTTCTCTATC | (Sac I) |
| AgeI-R | CT *GCTAGC*TATAGTTCTAGAGGTATCGGTTGTTTCGAGCTTATAG | (Nhe I) |
| ApaI-F | GCCTTGAGGGGCCTCCGGGGACGGCCCTTTGTGCGGGG | |
| ApaI-R | CCC CGCACAAAGGGGCCGTCCCGGAGCCCCCTCAAGGC | |

T means a replacement of G; C means a replacement of A; G means a replacement of T

**Supplemental Fig. S1. Construction of packaging plasmid psPAX2m-Pol carrying patient's HIV-1 pol gene.** The drug resistance Pol genes including the PR and RT from HIV-1 infected patients were individually cloned into the psPAX2m at Age I and Apa I restriction sites, which were designated as psPAX2m-Pol.

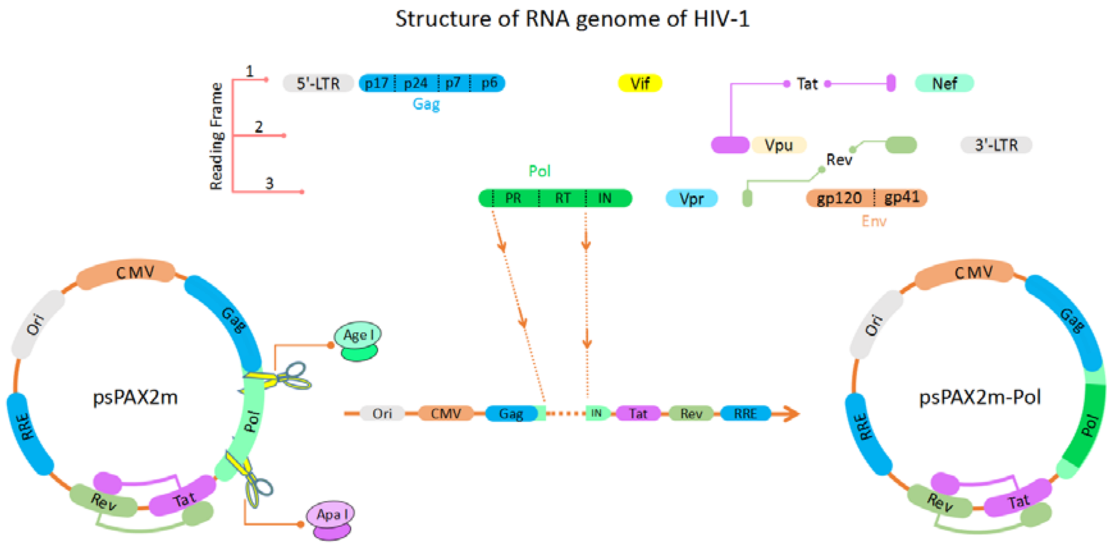

**Supplemental Fig. S2. Construction of transfer plasmid pHAGE-CMV-Luc-IRES-ZsGreen with dual-reporters.** EF1α promoter within plasmid of pHAGE-EF1α-IRES-ZsGreen was replaced by CMV promoter from pMD2.G at restriction sites Spe I and BamH I. The luciferase gene fragment from pGL3-Promoter Vector was sub-cloned into pHAGE-CMV-IRES-ZsGreen next to the IRES. The transfer vector with dual-reporter was designated pHAGE-CMV-Luc-IRES-ZsGreen.

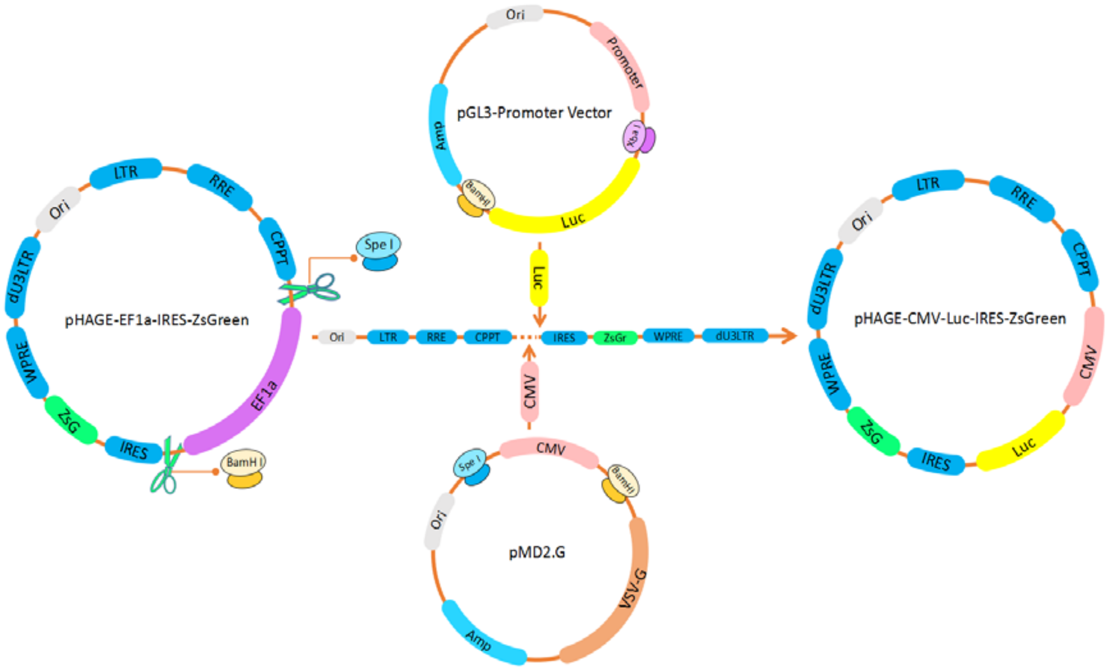

**Supplemental Fig. S3. Workflow of phenotypic drug susceptibility testing of HIV-1.**

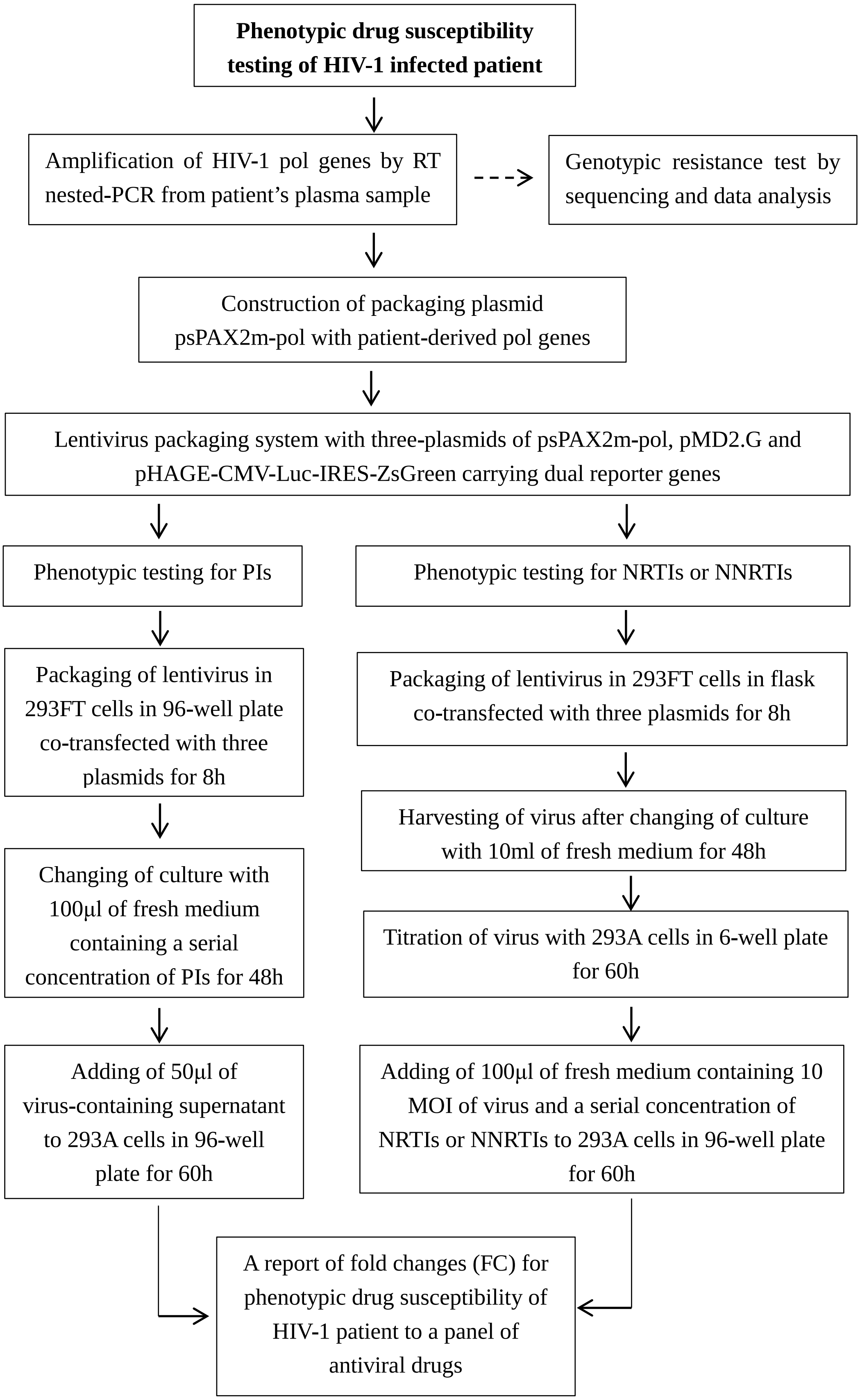

Supplement: Supplementary Information [file srep33559-s1.pdf]
